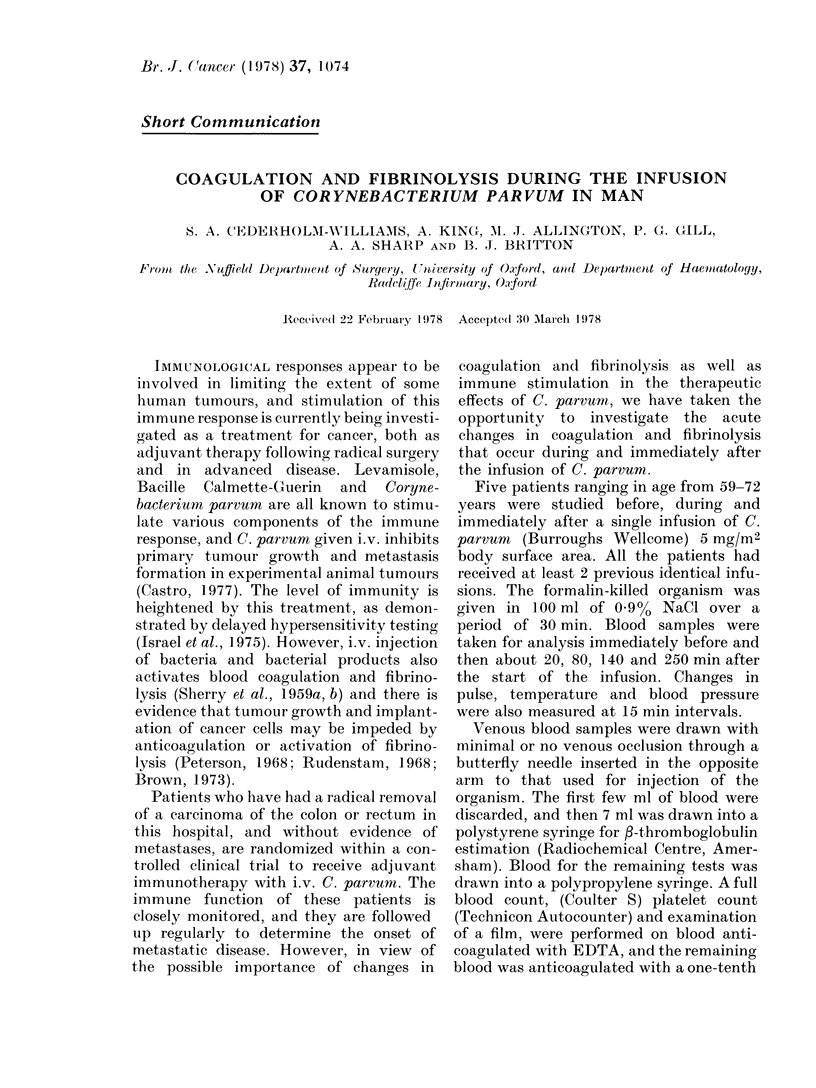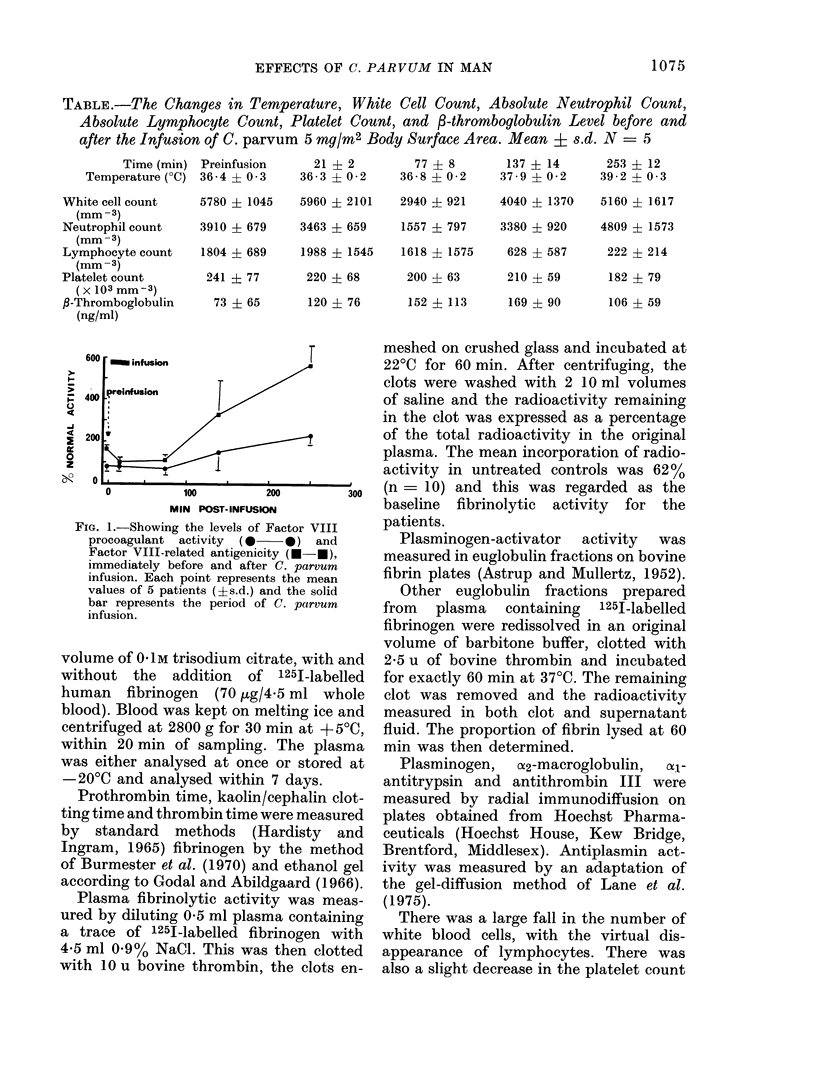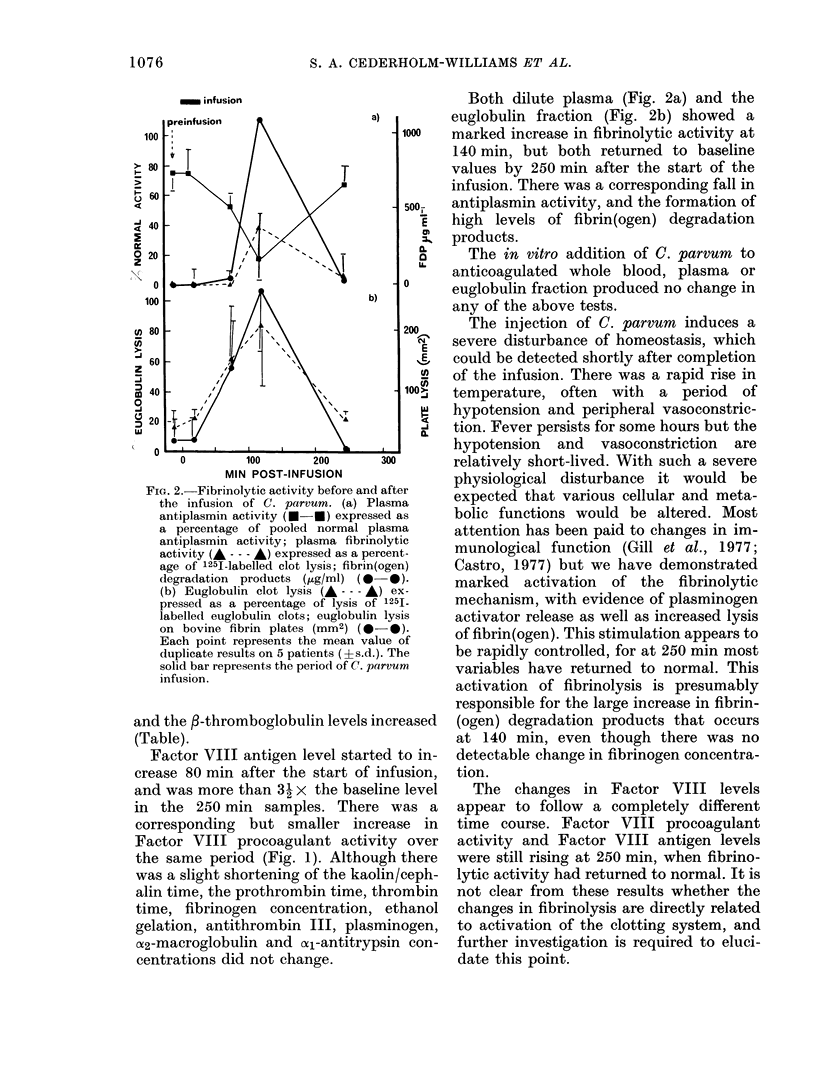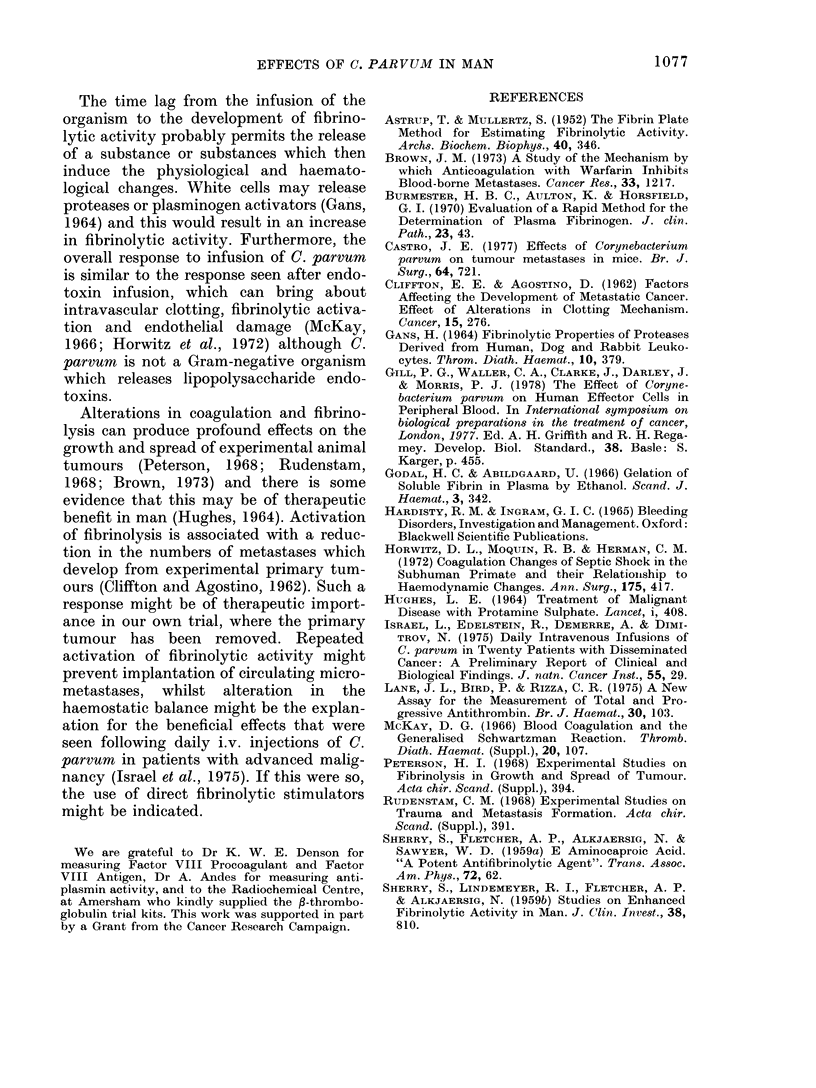# Coagulation and fibrinolysis during the infusion of Corynebacterium parvum in man.

**DOI:** 10.1038/bjc.1978.155

**Published:** 1978-06

**Authors:** S. A. Cederholm-Williams, A. King, M. J. Allington, P. G. Gill, A. A. Sharp, B. J. Britton


*Br. J. Cancer* (1978) **37**, 1074

***Short Communication***

### COAGULATION AND FIBRINOLYSIS DURING THE INFUSION OF *CORYNEBACTERIUM PARVUM* IN MAN

S. A. CEDERHOLM-WILLIAMS, A. KING, M. J. ALLINGTON, P. G. GILL, A. A. SHARP AND B. J. BRITTON

*From the Nuffield Department of Surgery, University of Oxford, and Department of Haematology, Radcliffe Infirmary, Oxford*

Received 22 February 1978 Accepted 30 March 1978

IMMUNOLOGICAL responses appear to be involved in limiting the extent of some human tumours, and stimulation of this immune response is currently being investigated as a treatment for cancer, both as adjuvant therapy following radical surgery and in advanced disease. Levamisole, Bacille Calmette-Guerin and *Corynebacterium parvum* are all known to stimulate various components of the immune response, and *C. parvum* given i.v. inhibits primary tumour growth and metastasis formation in experimental animal tumours (Castro, 1977). The level of immunity is heightened by this treatment, as demonstrated by delayed hypersensitivity testing (Israel *et al.*, 1975). However, i.v. injection of bacteria and bacterial products also activates blood coagulation and fibrinolysis (Sherry *et al.*, 1959*a*, *b*) and there is evidence that tumour growth and implantation of cancer cells may be impeded by anticoagulation or activation of fibrinolysis (Peterson, 1968; Rudenstam, 1968; Brown, 1973).

Patients who have had a radical removal of a carcinoma of the colon or rectum in this hospital, and without evidence of metastases, are randomized within a controlled clinical trial to receive adjuvant immunotherapy with i.v. *C. parvum*. The immune function of these patients is closely monitored, and they are followed up regularly to determine the onset of metastatic disease. However, in view of the possible importance of changes in coagulation and fibrinolysis as well as immune stimulation in the therapeutic effects of *C. parvum*, we have taken the opportunity to investigate the acute changes in coagulation and fibrinolysis that occur during and immediately after the infusion of *C. parvum*.

Five patients ranging in age from 59–72 years were studied before, during and immediately after a single infusion of *C. parvum* (Burroughs Wellcome) 5 mg/m$^2$ body surface area. All the patients had received at least 2 previous identical infusions. The formalin-killed organism was given in 100 ml of 0·9% NaCl over a period of 30 min. Blood samples were taken for analysis immediately before and then about 20, 80, 140 and 250 min after the start of the infusion. Changes in pulse, temperature and blood pressure were also measured at 15 min intervals.

Venous blood samples were drawn with minimal or no venous occlusion through a butterfly needle inserted in the opposite arm to that used for injection of the organism. The first few ml of blood were discarded, and then 7 ml was drawn into a polystyrene syringe for $\beta$-thromboglobulin estimation (Radiochemical Centre, Amersham). Blood for the remaining tests was drawn into a polypropylene syringe. A full blood count, (Coulter S) platelet count (Technicon Autocounter) and examination of a film, were performed on blood anticoagulated with EDTA, and the remaining blood was anticoagulated with a one-tenth

TABLE.—*The Changes in Temperature, White Cell Count, Absolute Neutrophil Count, Absolute Lymphocyte Count, Platelet Count, and β-thromboglobulin Level before and after the Infusion of C.* parvum *5 mg/m² Body Surface Area. Mean ± s.d. N = 5*

| Time (min) | Preinfusion | 21 ± 2 | 77 ± 8 | 137 ± 14 | 253 ± 12 |
|---|---|---|---|---|---|
| Temperature (°C) | 36·4 ± 0·3 | 36·3 ± 0·2 | 36·8 ± 0·2 | 37·9 ± 0·2 | 39·2 ± 0·3 |
| White cell count ($mm^{-3}$) | 5780 ± 1045 | 5960 ± 2101 | 2940 ± 921 | 4040 ± 1370 | 5160 ± 1617 |
| Neutrophil count ($mm^{-3}$) | 3910 ± 679 | 3463 ± 659 | 1557 ± 797 | 3380 ± 920 | 4809 ± 1573 |
| Lymphocyte count ($mm^{-3}$) | 1804 ± 689 | 1988 ± 1545 | 1618 ± 1575 | 628 ± 587 | 222 ± 214 |
| Platelet count ($\times 10^3$ $mm^{-3}$) | 241 ± 77 | 220 ± 68 | 200 ± 63 | 210 ± 59 | 182 ± 79 |
| β-Thromboglobulin (ng/ml) | 73 ± 65 | 120 ± 76 | 152 ± 113 | 169 ± 90 | 106 ± 59 |

FIG. 1.—Showing the levels of Factor VIII procoagulant activity (●——●) and Factor VIII-related antigenicity (■—■), immediately before and after *C. parvum* infusion. Each point represents the mean values of 5 patients (±s.d.) and the solid bar represents the period of *C. parvum* infusion.

volume of 0·1M trisodium citrate, with and without the addition of $^{125}$I-labelled human fibrinogen (70 μg/4·5 ml whole blood). Blood was kept on melting ice and centrifuged at 2800 g for 30 min at +5°C, within 20 min of sampling. The plasma was either analysed at once or stored at −20°C and analysed within 7 days.

Prothrombin time, kaolin/cephalin clotting time and thrombin time were measured by standard methods (Hardisty and Ingram, 1965) fibrinogen by the method of Burmester *et al.* (1970) and ethanol gel according to Godal and Abildgaard (1966).

Plasma fibrinolytic activity was measured by diluting 0·5 ml plasma containing a trace of $^{125}$I-labelled fibrinogen with 4·5 ml 0·9% NaCl. This was then clotted with 10 u bovine thrombin, the clots enmeshed on crushed glass and incubated at 22°C for 60 min. After centrifuging, the clots were washed with 2 10 ml volumes of saline and the radioactivity remaining in the clot was expressed as a percentage of the total radioactivity in the original plasma. The mean incorporation of radioactivity in untreated controls was 62% (n = 10) and this was regarded as the baseline fibrinolytic activity for the patients.

Plasminogen-activator activity was measured in euglobulin fractions on bovine fibrin plates (Astrup and Mullertz, 1952).

Other euglobulin fractions prepared from plasma containing $^{125}$I-labelled fibrinogen were redissolved in an original volume of barbitone buffer, clotted with 2·5 u of bovine thrombin and incubated for exactly 60 min at 37°C. The remaining clot was removed and the radioactivity measured in both clot and supernatant fluid. The proportion of fibrin lysed at 60 min was then determined.

Plasminogen, $\alpha_2$-macroglobulin, $\alpha_1$-antitrypsin and antithrombin III were measured by radial immunodiffusion on plates obtained from Hoechst Pharmaceuticals (Hoechst House, Kew Bridge, Brentford, Middlesex). Antiplasmin activity was measured by an adaptation of the gel-diffusion method of Lane *et al.* (1975).

There was a large fall in the number of white blood cells, with the virtual disappearance of lymphocytes. There was also a slight decrease in the platelet count

FIG. 2.—Fibrinolytic activity before and after the infusion of *C. parvum*. (a) Plasma antiplasmin activity (■—■) expressed as a percentage of pooled normal plasma antiplasmin activity; plasma fibrinolytic activity (▲ - - - ▲) expressed as a percentage of $^{125}$I-labelled clot lysis; fibrin(ogen) degradation products (μg/ml) (●—●). (b) Euglobulin clot lysis (▲ - - - ▲) expressed as a percentage of lysis of $^{125}$I-labelled euglobulin clots; euglobulin lysis on bovine fibrin plates (mm$^2$) (●—●). Each point represents the mean value of duplicate results on 5 patients (±s.d.). The solid bar represents the period of *C. parvum* infusion.

and the β-thromboglobulin levels increased (Table).

Factor VIII antigen level started to increase 80 min after the start of infusion, and was more than $3\frac{1}{2}\times$ the baseline level in the 250 min samples. There was a corresponding but smaller increase in Factor VIII procoagulant activity over the same period (Fig. 1). Although there was a slight shortening of the kaolin/cephalin time, the prothrombin time, thrombin time, fibrinogen concentration, ethanol gelation, antithrombin III, plasminogen, $\alpha_2$-macroglobulin and $\alpha_1$-antitrypsin concentrations did not change.

Both dilute plasma (Fig. 2a) and the euglobulin fraction (Fig. 2b) showed a marked increase in fibrinolytic activity at 140 min, but both returned to baseline values by 250 min after the start of the infusion. There was a corresponding fall in antiplasmin activity, and the formation of high levels of fibrin(ogen) degradation products.

The *in vitro* addition of *C. parvum* to anticoagulated whole blood, plasma or euglobulin fraction produced no change in any of the above tests.

The injection of *C. parvum* induces a severe disturbance of homeostasis, which could be detected shortly after completion of the infusion. There was a rapid rise in temperature, often with a period of hypotension and peripheral vasoconstriction. Fever persists for some hours but the hypotension and vasoconstriction are relatively short-lived. With such a severe physiological disturbance it would be expected that various cellular and metabolic functions would be altered. Most attention has been paid to changes in immunological function (Gill *et al.*, 1977; Castro, 1977) but we have demonstrated marked activation of the fibrinolytic mechanism, with evidence of plasminogen activator release as well as increased lysis of fibrin(ogen). This stimulation appears to be rapidly controlled, for at 250 min most variables have returned to normal. This activation of fibrinolysis is presumably responsible for the large increase in fibrin(ogen) degradation products that occurs at 140 min, even though there was no detectable change in fibrinogen concentration.

The changes in Factor VIII levels appear to follow a completely different time course. Factor VIII procoagulant activity and Factor VIII antigen levels were still rising at 250 min, when fibrinolytic activity had returned to normal. It is not clear from these results whether the changes in fibrinolysis are directly related to activation of the clotting system, and further investigation is required to elucidate this point.

The time lag from the infusion of the organism to the development of fibrinolytic activity probably permits the release of a substance or substances which then induce the physiological and haematological changes. White cells may release proteases or plasminogen activators (Gans, 1964) and this would result in an increase in fibrinolytic activity. Furthermore, the overall response to infusion of *C. parvum* is similar to the response seen after endotoxin infusion, which can bring about intravascular clotting, fibrinolytic activation and endothelial damage (McKay, 1966; Horwitz *et al.*, 1972) although *C. parvum* is not a Gram-negative organism which releases lipopolysaccharide endotoxins.

Alterations in coagulation and fibrinolysis can produce profound effects on the growth and spread of experimental animal tumours (Peterson, 1968; Rudenstam, 1968; Brown, 1973) and there is some evidence that this may be of therapeutic benefit in man (Hughes, 1964). Activation of fibrinolysis is associated with a reduction in the numbers of metastases which develop from experimental primary tumours (Cliffton and Agostino, 1962). Such a response might be of therapeutic importance in our own trial, where the primary tumour has been removed. Repeated activation of fibrinolytic activity might prevent implantation of circulating micrometastases, whilst alteration in the haemostatic balance might be the explanation for the beneficial effects that were seen following daily i.v. injections of *C. parvum* in patients with advanced malignancy (Israel *et al.*, 1975). If this were so, the use of direct fibrinolytic stimulators might be indicated.

We are grateful to Dr K. W. E. Denson for measuring Factor VIII Procoagulant and Factor VIII Antigen, Dr A. Andes for measuring antiplasmin activity, and to the Radiochemical Centre, at Amersham who kindly supplied the β-thromboglobulin trial kits. This work was supported in part by a Grant from the Cancer Research Campaign.

### REFERENCES

ASTRUP, T. & MULLERTZ, S. (1952) The Fibrin Plate Method for Estimating Fibrinolytic Activity. *Archs. Biochem. Biophys.*, **40**, 346.

BROWN, J. M. (1973) A Study of the Mechanism by which Anticoagulation with Warfarin Inhibits Blood-borne Metastases. *Cancer Res.*, **33**, 1217.

BURMESTER, H. B. C., AULTON, K. & HORSFIELD, G. I. (1970) Evaluation of a Rapid Method for the Determination of Plasma Fibrinogen. *J. clin. Path.*, **23**, 43.

CASTRO, J. E. (1977) Effects of *Corynebacterium parvum* on tumour metastases in mice. *Br. J. Surg.*, **64**, 721.

CLIFFTON, E. E. & AGOSTINO, D. (1962) Factors Affecting the Development of Metastatic Cancer. Effect of Alterations in Clotting Mechanism. *Cancer*, **15**, 276.

GANS, H. (1964) Fibrinolytic Properties of Proteases Derived from Human, Dog and Rabbit Leukocytes. *Throm. Diath. Haemat.*, **10**, 379.

GILL, P. G., WALLER, C. A., CLARKE, J., DARLEY, J. & MORRIS, P. J. (1978) The Effect of *Corynebacterium parvum* on Human Effector Cells in Peripheral Blood. In *International symposium on biological preparations in the treatment of cancer, London, 1977.* Ed. A. H. Griffith and R. H. Regamey. Develop. Biol. Standard., **38**. Basle: S. Karger, p. 455.

GODAL, H. C. & ABILDGAARD, U. (1966) Gelation of Soluble Fibrin in Plasma by Ethanol. *Scand. J. Haemat.*, **3**, 342.

HARDISTY, R. M. & INGRAM, G. I. C. (1965) Bleeding Disorders, Investigation and Management. Oxford: Blackwell Scientific Publications.

HORWITZ, D. L., MOQUIN, R. B. & HERMAN, C. M. (1972) Coagulation Changes of Septic Shock in the Subhuman Primate and their Relationship to Haemodynamic Changes. *Ann. Surg.*, **175**, 417.

HUGHES, L. E. (1964) Treatment of Malignant Disease with Protamine Sulphate. *Lancet*, i, 408.

ISRAEL, L., EDELSTEIN, R., DEMERRE, A. & DIMITROV, N. (1975) Daily Intravenous Infusions of *C. parvum* in Twenty Patients with Disseminated Cancer: A Preliminary Report of Clinical and Biological Findings. *J. natn. Cancer Inst.*, **55**, 29.

LANE, J. L., BIRD, P. & RIZZA, C. R. (1975) A New Assay for the Measurement of Total and Progressive Antithrombin. *Br. J. Haemat.*, **30**, 103.

McKAY, D. G. (1966) Blood Coagulation and the Generalised Schwartzman Reaction. *Thromb. Diath. Haemat.* (Suppl.), **20**, 107.

PETERSON, H. I. (1968) Experimental Studies on Fibrinolysis in Growth and Spread of Tumour. *Acta chir. Scand.* (Suppl.), 394.

RUDENSTAM, C. M. (1968) Experimental Studies on Trauma and Metastasis Formation. *Acta chir. Scand.* (Suppl.), 391.

SHERRY, S., FLETCHER, A. P., ALKJAERSIG, N. & SAWYER, W. D. (1959*a*) E Aminocaproic Acid. "A Potent Antifibrinolytic Agent". *Trans. Assoc. Am. Phys.*, **72**, 62.

SHERRY, S., LINDEMEYER, R. I., FLETCHER, A. P. & ALKJAERSIG, N. (1959*b*) Studies on Enhanced Fibrinolytic Activity in Man. *J. Clin. Invest.*, **38**, 810.